# Grass Carp Prx 3 Elevates Host Antioxidant Activity and Induces Autophagy to Inhibit Grass Carp Reovirus (GCRV) Replication

**DOI:** 10.3390/antiox11101952

**Published:** 2022-09-29

**Authors:** Xinyu Liang, Yongming Li, Pengfei Chu, Qian Wang, Hanyue Wang, Lanjie Liao, Cheng Yang, Zuoyan Zhu, Yaping Wang, Libo He

**Affiliations:** 1 State Key Laboratory of Freshwater Ecology and Biotechnology, Institute of Hydrobiology, Chinese Academy of Sciences, Wuhan 430072, China; 2 University of Chinese Academy of Sciences, Beijing 100049, China; 3 College of Animal Science and Technology, Yangzhou University, Yangzhou 225009, China; 4 Innovative Academy of Seed Design, Chinese Academy of Sciences, Beijing 100101, China

**Keywords:** grass carp, peroxiredoxin 3, grass carp reovirus, virus replication, ROS, antioxidant, autophagy

## Abstract

Peroxiredoxins are a family of antioxidant proteins that protect cells from oxidative damage caused by reactive oxygen species (ROS). Herein, the peroxiredoxin 3 gene from grass carp (*Ctenopharyngodon idellus*), named *CiPrx3*, was cloned and analyzed. The full-length cDNA of *CiPrx3* is 1068 bp long, with a 753 bp open reading frame (ORF) that contains a thioredoxin-2 domain, two peroxiredoxin signature motifs, and two highly conserved cysteine residues. *CiPrx3* was ubiquitously expressed in all the tested tissues, while its expression level was altered significantly after exposure to grass carp reovirus (GCRV) and pathogen-associated molecular patterns (PAMPs). *CiPrx3* was localized in the mitochondria of transfected cells and concentrated in the nucleus after poly (I:C) treatment. Transformation of *CiPrx3* into *Escherichia coli* enhanced host resistance to H_2_O_2_ and heavy metals. Purified recombinant *CiPrx3* proteins could protect DNA against oxidative damage. Overexpression of *CiPrx3* in fish cells reduced intracellular ROS, increased cell viability, and decreased cell apoptosis caused by H_2_O_2_ stimulation and GCRV infection. Further study indicated that *CiPrx3* induced autophagy to inhibit GCRV replication in fish cells. Collectively, these results imply that grass carp Prx3 elevates host antioxidant activity and induces autophagy to inhibit GCRV replication.

## 1. Introduction

Peroxiredoxins (Prxs), also termed thioredoxin peroxidase (TPx), are a family of antioxidant enzymes. By scavenging reactive oxygen species (ROS), Prxs protect cells from oxidative damage [1]. Prxs are ubiquitously conserved in a wide variety of living organisms ranging from prokaryotes to eukaryotes [2,3,4,5]. The Prxs family can be classified into six subtypes based on their structure and catalytic domains: the typical 2-Cys type (Prx1, Prx2, Prx3, and Prx4), the atypical 2-Cys type (Prx5), and the 1-Cys type (Prx6) [6,7]. The first member of the Prxs family was discovered in the yeast *Saccharomyces cerevisiae* as an enzyme with a molecular weight of 25 kDa that protects cellular components from reactive sulfur species rather than ROS [8]. Moreover, Prxs family members were also reported to be involved in a variety of physiological processes, including cell growth, differentiation, embryonic development, immune response, apoptosis, lipid droplet-related metabolism, and ROS balance [9].

Peroxiredoxin 3 (Prx3) is a member of the typical 2-Cys peroxiredoxin family and is located exclusively in mitochondria. Previous studies have suggested that Prx3 is involved in maintaining the redox balance in mitochondria and acts as a key regulator of apoptosis [10,11]. For example, Prx3 levels establish a redox set point that is essential for proper mitochondrial structure, function, and cell cycle progression of mesothelioma cells [12]. Overexpression of Prx3 improves glucose tolerance in mice by reducing mitochondrial H_2_O_2_ [13]. Prx3 is an indispensable ROS scavenger that protects tumor cells against oxidative damage and subsequent apoptosis [14]. To date, the Prx3 gene has been cloned from several fish species, including medaka [15], rock bream [16], common carp [17], and big-belly seahorses [5]. However, the functions of Prx3 in teleost fish, especially the function in host immune response and defense against pathogens, are still unknown. Understanding the particular function of Prx3 in teleost fish will benefit fish disease control and prevention, as well as disease-resistant fish breeding programs.

Grass carp (*Ctenopharyngodon idellus*), a traditional aquaculture species in China with a history spanning over 60 years, occupies more than 21% of total freshwater aquaculture production. Grass carp is the most highly consumed freshwater fish in China, and its production has added up to approximately 5.50 million tons in recent years [18]. However, there are often disease outbreaks that induce huge economic losses to grass carp aquaculture. In particular, grass carp reovirus (GCRV) induced hemorrhagic disease is one of the most serious diseases that requires attention [19]. Nevertheless, no effective drugs or vaccines against GCRV have been found until now. Therefore, identification of grass carp genes involved in host immune defense is important for antiviral drug development or fish breeding programs.

In this study, the Prx3 gene was cloned from grass carp. The gene structure, expression profile, and localization pattern were analyzed. Moreover, the functions of Prx3 during antioxidant and GCRV infection were also investigated. Our results provide important information for further understanding the function of Prx3 in teleost fish.

## 2. Materials and Methods

### 2.1. Ethics Statement

All animal experiments complied with the ARRIVE guidelines and followed the Guide for the Care and Use of Laboratory Animals (State Scientific and Technological Commission of China, 2017). The protocol was approved by the Ethics Committee of the Institute of Hydrobiology, Chinese Academy of Sciences (CAS) (protocol code: IHB2020-0810, approval date: 10 August 2020). All surgery was performed under MS-222 anesthesia, and all efforts were made to minimize suffering.

### 2.2. Cell and Antibodies

Grass carp ovary (GCO) cells were cultured in M199 medium (HyClone, Logan, UT, USA) supplemented with 10% fetal bovine serum (FBS), 100 IU/mL penicillin, and 100 mg/mL streptomycin under humidified conditions with 5% CO_2_ at 28 °C. Mouse anti-β-actin antibody was purchased from Proteintech (Proteintech, Wuhan, China). Mouse anti-p62 antibody was purchased from Beyotime (Beyotime, Shanghai, China). Rabbit anti-LC3B antibody, horseradish peroxidase (HRP)-conjugated goat anti-rabbit IgG, and HRP-conjugated goat anti-mouse IgG were purchased from Sigma (Sigma, Saint Louis, MS, USA). Rabbit anti-NS80 and Rabbit anti-VP5 antibodies were prepared in our laboratory. Briefly, the complete ORF sequence of NS80 and VP5 was amplified and ligated into the pET-32a expression vector. The resulting plasmids (pET-32a-NS80 and pET-32a-VP5) were transformed into *E. coli* BL21 and cultured in 400 mL LB medium until an OD600 of 0.6 was reached. The cells were then induced with IPTG for 4 h at 30 °C in order to express the fusion protein. The fusion protein was purified using BeyoGold™ His-tag Purification Resin, mixed with an equal volume of Freund’s adjuvant (Sigma, Saint Louis, MS, USA), and thereafter used to immunize the rabbit. Anti-NS80 or anti-VP5 serum was collected after immunizing the rabbit three times.

### 2.3. GCRV Challenge and PAMP Stimulation

Healthy three-month-old grass carp with an average weight and length of 3–5 g and 5–8 cm were used in the study. All of the fish were obtained from a full-sib family bred by the Guanqiao Experimental Station, Institute of Hydrobiology, CAS. Before further processing, the fish were fed commercial feed (Tong Wei, Chengdu, China) twice a day and acclimatized in aerated fresh water at 28 °C for one week. Grass carp were used for further study after no abnormal symptoms were observed.

In accordance with our previous description, we conducted a virus challenge experiment [20]. Briefly, approximately 100 grass carp were intraperitoneally injected with GCRV in a volume of 200 μL (GCRV subtype II, 2.97 × 10^3^ RNA copies/μL). At 0–6 days post-infection (dpi), five fish individuals were collected, and spleen and liver samples were removed for analysis. Then, RNA was prepared to analyze the response of *CiPrx3* after GCRV infection. Moreover, five uninfected fish were selected, and samples from the skin, gill, spleen, muscle, brain, liver, intestine, heart, head kidney, and middle kidney were obtained. RNA from these tissues was prepared in order to analyze the tissue distribution of *CiPrx3*.

In addition, 150 grass carp were collected for pathogen-associated molecular patterns (PAMPs) stimulation. Fish were divided into three groups (50 per group) and then intraperitoneally injected with 200 μL PBS, 200 μL lipopolysaccharide (LPS) (0.5 mg/mL, dissolved in PBS), or 200 μL poly (I:C) (1 mg/mL, dissolved in PBS). At 3, 6, 12, 24, and 48 h post-injection (hpi), five fish individuals from each group were collected, and spleen and liver samples were removed for analysis. RNA from these tissues was prepared in order to analyze the response of *CiPrx3* after PAMP stimulation.

### 2.4. cDNA Cloning

Total RNA was isolated from the tissues of healthy grass carp using a TRIzol reagent (Invitrogen, Carlsbad, CA, USA). RNA concentration was measured using the Qubit RNA assay kit (Life Technologies, California, USA), and integrity was assessed with the RNA nano 6000 assay kit (Agilent Technologies, Santa Clara, CA, USA). First-strand cDNA was synthesized by using the ReverTra Ace kit (Toyobo, Osaka, Japan) and oligo (dT)-adaptor as primers. Specific primers (Table S1) were designed according to the cDNA sequences of zebrafish Prx3 (NM_001013460.3) and the deduced cDNA sequences of grass carp Prx3 to amplify the ORF sequence of *CiPrx3*. Moreover, the 5′ and 3′ untranslated regions (UTRs) of *CiPrx3* were obtained by rapid amplification of cDNA ends (RACE) using the 5′ and 3′ full RACE Kit (TaKaRa, Kusatsu, Japan). PCR products were inserted into pMD18-T vectors (Takara, Kusatsu, Japan) and transformed into *E. coli* DH5α for sequencing by a commercial company (TsingKe, Beijing, China). Finally, the full-length cDNA of *CiPrx3* was obtained by assembling the ORF sequence and 5′ and 3′ UTR sequences using DNAMAN software.

### 2.5. Sequence Analysis

BLAST (https://blast.ncbi.nlm.nih.gov/Blast.cgi) (accessed on 15 January 2022) was used to search for CiPrx3 homologues in the National Center for Biotechnology Information (NCBI). The Sequence Manipulation Suite (SMS) (http://www.bio-soft.net/sms) (accessed on 23 January 2022) [21] was used to analyze the nucleotide and predicted amino acid sequences of *CiPrx3*. Simple Modular Architecture Research Tool (SMART) (http://smart.embl-heidelberg.de) (accessed on 23 January 2022) [22] software was used to predict the functional domains of the amino acid sequence of *CiPrx3*. ClustalW2.1 (http://www.clustal.org/clustal2) (accessed on 23 January 2022) [23] was used to perform multiple sequence alignments of Prx3 among different species. MEGA 7.0 software (http://www.megasoftware.net/index.html) (accessed on 23 January 2022) [24] was used to construct the Maximum Likelihood (ML) phylogenetic tree on the basis of amino acid sequences, and the bootstrap values of the branches were obtained by testing the tree 10,000 times.

### 2.6. Gene Expression Analysis

Total RNA was isolated from ten tissues of five healthy grass carp and reverse transcribed to obtain cDNA as described above. RNA concentration and integrity were also determined as described previously. cDNA from the same tissues was mixed and served as the template for real-time quantitative PCR (RT-qPCR) analysis of the expression level of *CiPrx3* in the different tissues. RT-qPCR was performed using a fluorescence quantitative PCR instrument (Bio-Rad, Hercules, CA, USA). Each reaction mixture contained 0.8 μL forward and reverse primers (for each primer), 1 μL cDNA template, 10 μL 2 × SYBR qPCR Master Mix (Vazyme, Nanjing, China), and 7.4 μL ddH_2_O. Three replicates were included for each sample, and β-actin was used as an internal control to normalize the gene expression. The program was as follows: 95 °C for 10 s; 40 cycles of 95 °C for 15 s, 56 °C for 30 s, 72 °C for 30 s; and melt curve construction. The 2^−^^ΔΔCt^ method was employed in order to calculate the relative expression levels [25]. The specific primers for RT-qPCR are listed in Table S1.

Moreover, total RNA was isolated from liver and spleen tissues of grass carp collected on different days (0–6 days) post GCRV infection or at different time points (3, 6, 12, 24, and 48 h) after PAMP stimulation, and then reverse transcribed to obtain cDNA as described above. The cDNA from the same tissues was mixed and served as the template for RT-qPCR analysis of the response of *CiPrx3* after GCRV infection and PAMP stimulation. The program, reaction mixture, and primers for RT-qPCR were the same as above. 

### 2.7. Fluorescence Observation

The complete open reading frame (ORF) of *CiPrx3* was amplified and inserted into plasmids pEGFP-N3. Obtained plasmids (pEGFP-*CiPrx3*) were confirmed by DNA sequencing. GCO cells grown on coverslips in 6-well plates were transfected with pEGFP-*CiPrx3* using Lipofectamine™ 3000, according to the manufacturer’s instructions. The transfected cells were stimulated by LPS or poly (I:C) or not stimulated. After 24 h, cells were fixed with 4% paraformaldehyde and then stained with Hoechst 33342. Finally, the cells were mounted with 50% glycerol and observed under an UltraVIEW VOX confocal system (PerkinElmer, Fremont, CA, USA). Moreover, pDsRed2-Mito and pEGFP-*CiPrx3* were cotransfected into GCO cells to determine the precise localization of *CiPrx3*. The transfected cells were fixed and stained as described above and observed under the same confocal system.

### 2.8. Protein Expression and Purification

The complete ORF sequence of *CiPrx3* was amplified and then ligated into the pEASY-Blunt expression vector (TransGen, Beijing, China). The resulting plasmid (pEASY-*CiPrx3*) was transformed into the *E. coli* BL21 (DE3) strain, and the bacterium was induced for 10 h with 1 mM IPTG at 20 °C to express the fusion protein. Then, cells were collected and the fusion protein was purified with NI-NTA resin according to the manufacturer’s protocols, as described previously [26]. The concentration of the purified protein was determined by the BCA Protein Assay Kit (Novagen, Hilden, Germany). Moreover, Western blotting was performed in order to confirm the correctness of the purified fusion protein. An anti-His tag antibody (Proteintech, Wuhan, China) was used as the primary antibody at a 1:1000 dilution, followed by HRP-conjugated goat anti-rabbit IgG (Tiangen, Beijing, China) at a 1:5000 dilution as the secondary antibody. Finally, the immunoblot signals were detected using an HRP-DAB Detection Kit (Tiangen, Beijing, China).

### 2.9. Mixed-Function Oxidase Assay

The mixed-function oxidase (MFO) assay was carried out as described previously [26] in order to determine the degree of DNA breakage caused by the ROS generated from the thiol/Fe^3+^/O_2_^−^ MFO system and to assess whether *CiPrx3* could protect the DNA from breakage. A total reaction volume of 50 μL mixtures (10 μM FeCl_3_, 10 mM DTT, and different concentrations of purified r*CiPrx3* fusion protein) was incubated at 37 °C for 1 h. Then, 1 μg of pcDNA3.1 supercoiled DNA was added to each reaction mixture and incubated further at 37 °C for another 1 h. Finally, the reaction mixtures were analyzed using a 1% agarose gel, stained with ethidium bromide, and then visualized under UV light.

### 2.10. Antioxidant Activity and Heavy Metal-Resistant Ability Assay

*E. coli* BL21 cells were transformed with pEASY-CiPrx3 or pEASY-Blunt and then cultured in 5 mL LB with ampicillin at 37 °C (220 rpm) until the OD_600_ reached approximately 0.6. Then, the cultures were induced with IPTG for 3 h. The agarose plates were coated with 200 μL *E. coli* BL21 cells containing pEASY-*CiPrx3* or pEASY-Blunt. Sterile filter papers (diameter: 6 mm) were soaked with heavy metal ions (Copper, Zinc, Chromium, and Ferrum) at a concentration of 1 mol/L or soaked with H_2_O_2_ at different concentrations (30%, 20%, 15%, 10%, 6%, and 3%) and then placed on agarose plates which were cultured overnight. The inhibition zone diameters between the two *E. coli* BL21 strains were measured and compared.

Moreover, we also analyzed the antioxidant activity of *CiPrx3* in fish cells. The ORF sequence of *CiPrx3* was amplified and fused with the HA tag and then inserted into pcDNA3.1 vector to obtain plasmid pcDNA3.1-*CiPrx3*. GCO cells were transfected with pcDNA3.1 or pcDNA3.1-*CiPrx3* and then treated with H_2_O_2_ or infected with GCRV. Cells were harvested at different time points after treatment/infection. Cell viability was examined by CCK-8 assay. Cell apoptosis and intracellular ROS were detected by flow cytometry with an ANNEXIN V-FITC/PI Apoptosis Assay kit (ZomanBio, Beijing, China) and a reactive oxygen species assay kit (Beyotime, Shanghai, China).

### 2.11. Anti-Viral Effect Analysis

In order to analyze the role of *CiPrx3* during GCRV infection, GCO cells were transfected with pcDNA3.1 or pcDNA3.1-*CiPrx3* and then infected with GCRV at a multiplicity of infection (MOI) of 1. Cells were harvested at different time points (12, 24, and 36 h) post-infection. The relative mRNA expression levels of genes encoding the GCRV nonstructural protein NS80 or structural proteins VP5 and VP7 were determined by RT-qPCR. Western blotting was also performed to examine the relative protein expression levels of NS80 and VP5. Rabbit anti-NS80 and Rabbit anti-VP5 antibodies were used as the primary antibodies at a 1:1000 dilution, followed by HRP-conjugated goat anti-rabbit IgG at a 1:5000 dilution as the secondary antibody.

### 2.12. Autophagy Level Detection

Plasmid pEGFP-LC3B, which contained the autophagy report gene LC3B, was constructed as described previously [27]. pEGFP-LC3B was cotransfected with pcDNA3.1 or pcDNA3.1-*CiPrx3* into GCO cells and collected at 24 h post-transfection. Cells were subjected to fluorescence observation in order to examine the GFP-LC3B puncta in transfected cells. Furthermore, GCO cells were transfected with pcDNA3.1 or pcDNA3.1-*CiPrx3* and then infected with 1 MOI of GCRV. Cells were harvested at different time points post-infection, and protein expression levels of LC3-II/LC3-I and autophagy substrate P62 were determined by Western blotting. Rabbit anti-LC3B antibodies and mouse anti-p62 antibodies were used as the primary antibodies, followed by HRP-conjugated goat anti-rabbit IgG and HRP-conjugated goat anti-mouse IgG as the secondary antibody,.

### 2.13. Statistical Analysis

All experimental data were analyzed by one-way variance (ANOVA) through SPSS (version 16.0; IBM Corporation, Armonk, NY, USA). Differences were considered significant and extremely significant at *p* ≤ 0.05 and *p* ≤ 0.01, respectively. *p* ≤ 0.05 and *p* ≤ 0.01 were denoted by * and **, respectively. 

## 3. Results

### 3.1. Cloning and Characterization of Grass Carp Peroxiredoxin 3

The full-length cDNA of the grass carp peroxiredoxin 3 gene (*CiPrx3*) was obtained by PCR and RACE. The full-length cDNA of *CiPrx3* is 1068 long, with a 753 bp ORF, a 13 bp 5′ UTR, and a 302 bp 3′ UTR (Figure S1A). Structural prediction of the deduced amino acid sequence of *CiPrx3* was carried out by SMART software. The results showed that the *CiPrx3* protein sequence contained a thioredoxin-2 domain, two peroxiredoxin signature motifs (FYPLDFTFVCPTEI and GEVCFA), and two highly conserved cysteine residues (Cys103 and Cys224) (Figure S1B).

**Figure 1:**
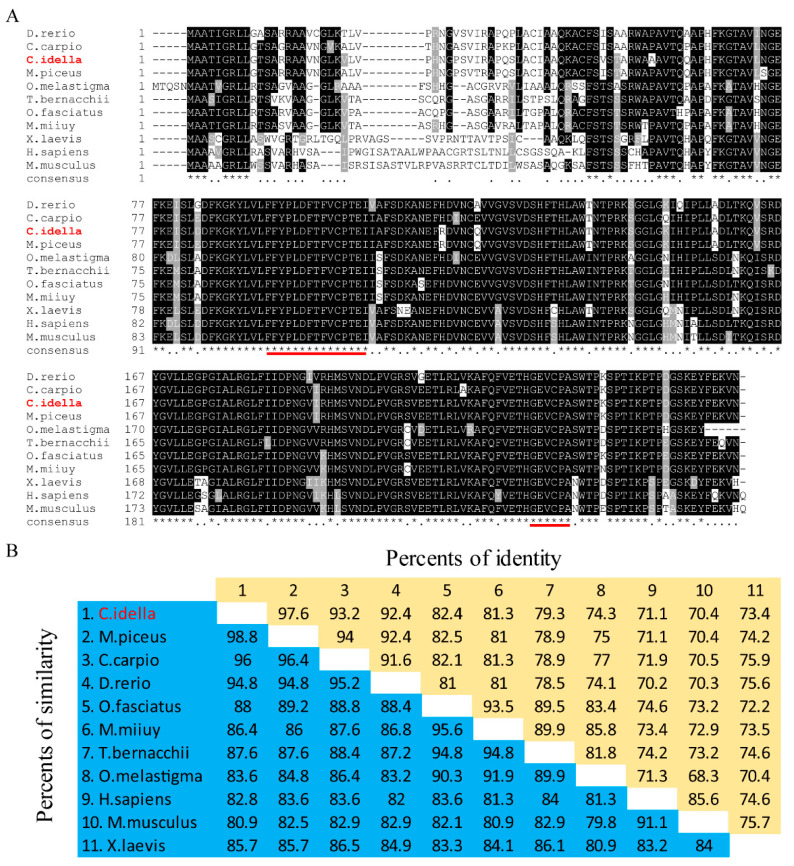
Multiple sequence alignment of *CiPrx3* with its homologues from other species. (**A**) Multiple sequence analysis of *CiPrx3* by ClustalW. The numbers of amino acids are listed on the left side of alignments. The black shade represents 100% identity, while dark gray represents 80% identity. Prxs signature motifs FYPLDFTFVCPTEI and GEVCPA are marked with red lines. (**B**) Identity and similarity of *CiPrx3* with its counterparts from other species. The percent identity is shown in the upper triangle, and the percent similarity is shown in the lower triangle. GenBank accession numbers for the protein sequences are as follows. *Mylopharyngodon piceus* Prx3 (ALD62539.1); *Trematomus bernacchii* Prx3 (APG79659.1); *Oplegnathus fasciatus* Prx3 (AJC98155.1); *Miichthys miiuy* Prx3 (AGT56738.1); *Cyprinus carpio* Prx3 (ALG02339.1); *Homo sapiens* Prx3 (AAH08435.1); *Mus musculus* Prx3 (EDL01849.1); *Danio rerio* Prx3 (AAH92846.1); *Oryzias melastigma* Prx3 (AEA51070.1); *Xenopus laevis* Prx3 (NP 001086130.1).

Multiple alignments of the deduced amino acid sequence of *CiPrx3,* with its homologs in other fishes and mammals, indicated that Prx3 proteins were highly conserved in both fish and mammals, especially the two peroxiredoxin signature motifs (FYPLDFTFVCPTEI and GEVCFA) (Figure 1A). Moreover, pairwise sequence alignment revealed that *CiPrx3* shares 70.4–97.6% protein sequence identity with its homologs from other species, in which *CiPrx3* showed the most similar identity to that of *Mylopharyngodon piceus* (97.6% sequence similarity), followed by *Cyprinus carpio* and *Danio rerio* (93.2% and 92.4% sequence similarity, respectively) (Figure 1B).

**Figure 2:**
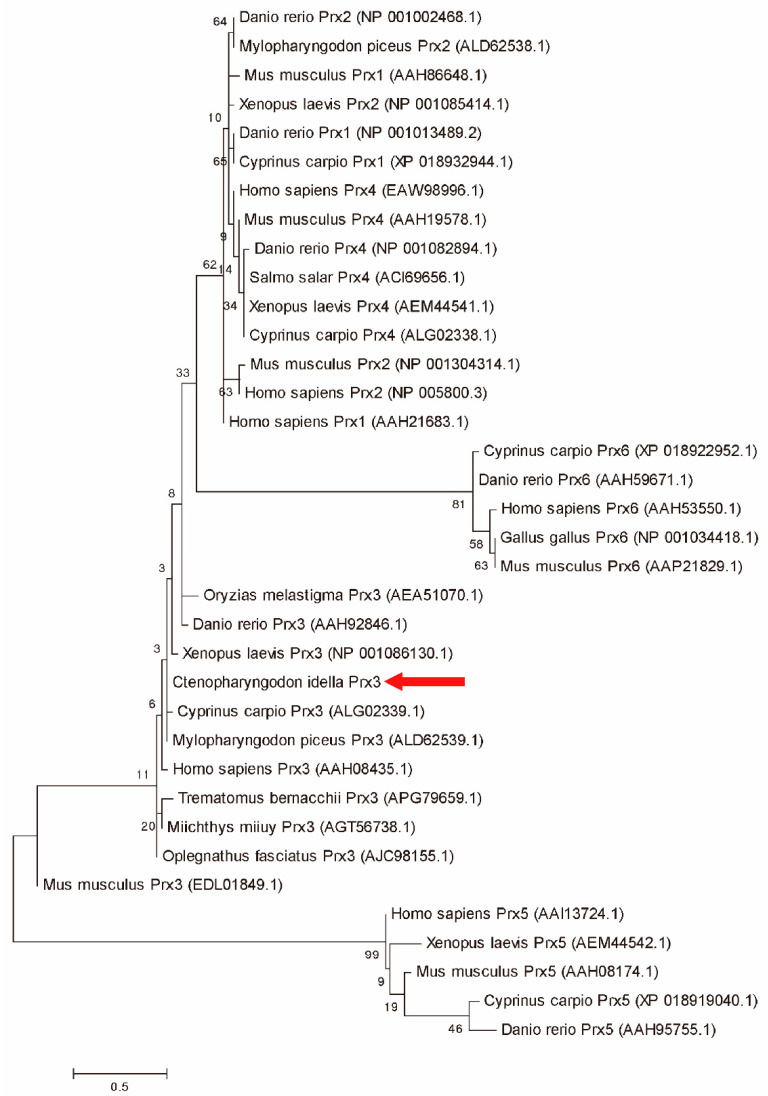
Maximum Likelihood (ML) phylogenetic tree analyses of *CiPrx3* with other Prx proteins from other species. The confidence in each node was assessed by 10,000 bootstraps.

In order to determine the molecular evolutionary relationship of *CiPrx3*, a phylogenetic tree was constructed by using Prx protein sequences from various vertebrate species. As shown in Figure 2, the phylogenetic tree could be divided into three branches, which correspond to the typical 2-Cys Prxs, atypical 2-Cys Prxs, and 1-Cys Prxs. *CiPrx3* was located in the branch of typical 2-Cys Prxs and closely related to that of *C. carpio* and *M. piceus* (Figure 2).

### 3.2. Expression Patterns of CiPrx3

**Figure 3:**
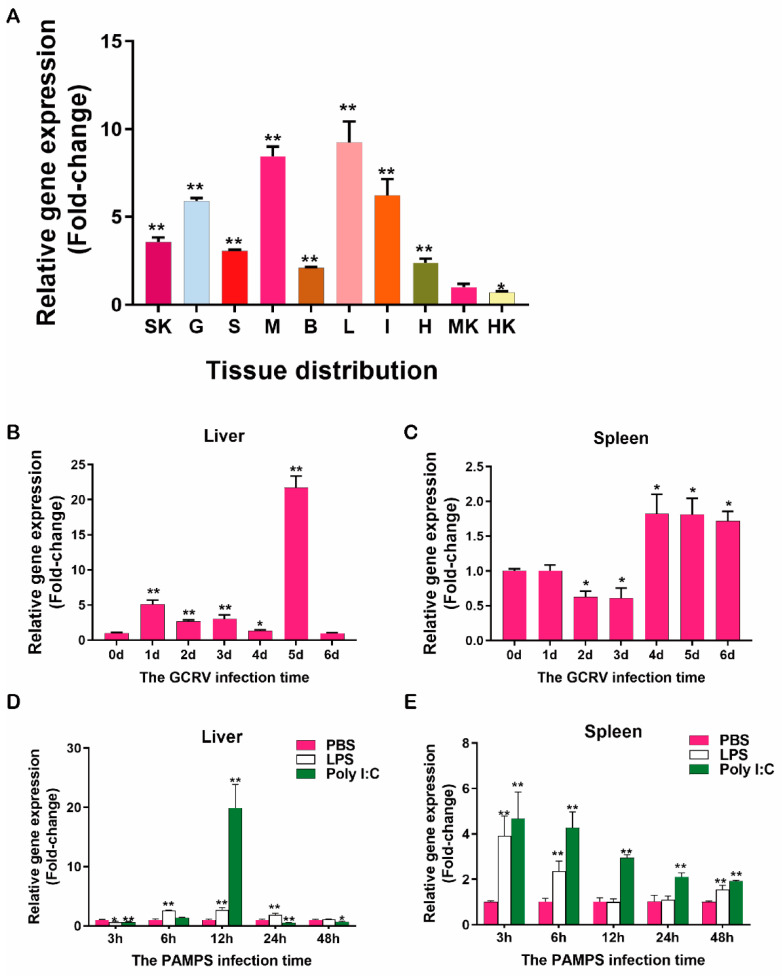
Expression patterns of *CiPrx3*. (**A**) Tissue distribution of *CiPrx3*. The determined tissues included SK: skin; S: spleen; B: brain; G: gill; L: liver; HK: head kidney; H: heart; MK: middle kidney; M: muscle; and I: intestine (*n* = 5). The relative expression is the ratio of gene expression in different tissues relative to that in the middle kidney. (**B**,**C**) Expression level of *CiPrx3* in the liver (**B**) and spleen (**C**) after GCRV infection. The relative expression is the ratio of gene expression after exposure to GCRV (1, 2, 3, 4, 5, and 6 days) to that in the control group (0 day) in the same tissue. (**D**,**E**) Expression pattern of *CiPrx3* in the liver (**D**) and spleen (**E**) after PAMP stimulation. The relative expression was calculated as the ratio of the gene expression level in the LPS- and poly (I:C)-treated groups relative to that in the PBS-treated group at the same time point. The β-actin was used as an internal control. All data are given in terms of relative mRNA expression as the mean ± SD. Asterisks represent significant differences (* = *p* ≤ 0.05, ** = *p* ≤ 0.01).

RT-qPCR was carried out to study the tissue distribution of *CiPrx3* in healthy grass carp. Using the middle kidney as a baseline, mRNA expression levels of *CiPrx3* in other tissues were expressed as fold changes relative to the middle kidney expression level. As shown in Figure 3A, *CiPrx3* was ubiquitously expressed in all ten tested tissues, while the expression level differed among these different tissues. Relatively high expression levels of *CiPrx3* were detected in the liver (9.25-fold), muscle (8.45-fold), intestine (6.22-fold), and gill (5.91-fold), whereas relatively low expression levels were detected in the middle kidney (1.00-fold) and head kidney (0.70-fold).

The total RNA from the liver and spleen of grass carp at 0–6 days post GCRV infection was reverse transcribed, then used for RT-qPCR analysis to determine the response of *CiPrx3.* In the liver, the mRNA expression level of *CiPrx3* was increased at 1–5 dpi, reached the peak level at 5 dpi (21.75-fold), then declined to the baseline level at 6 dpi (0.98-fold) (Figure 3B). The mRNA expression pattern of *CiPrx3* in the spleen was different from that observed in the liver. Specifically, the *CiPrx3* expression level was unchanged at 1 dpi (1.00-fold), and decreased at 2 dpi (0.62-fold) and 3 dpi (0.61-fold), followed by an increase at 4–6 dpi (1.72–1.82-fold) (Figure 3C).

Moreover, the expression of *CiPrx3* was analyzed after PAMP stimulation. As shown in Figure 3D,E, *CiPrx3* showed different response patterns in the liver and spleen after LPS and poly (I:C) stimulation. In the liver, LPS stimulation induced the downregulation of *CiPrx3* at 3 hpi (0.67-fold), and then upregulated *CiPrx3* at 6 hpi (2.58-fold), 12 hpi (2.71-fold), and 24 hpi (1.84-fold). The expression level did not change at 48 hpi (1.12-fold). Poly (I:C) stimulation resulted in dramatic upregulation of *CiPrx3* expression levels at 12 hpi (19.90-fold), whereas its expression level was decreased or not changed at other time points (Figure 3D). In the spleen, the *CiPrx3* expression level increased at 3 hpi (3.92-fold), 6 hpi (2.35-fold), and 48 hpi (1.55-fold) and showed no significant change at 12 hpi (1.00-fold) and 24 hpi (1.09-fold) after LPS stimulation. Interestingly, the *CiPrx3* expression level was increased at all examined time points (1.93–4.67-fold) after poly (I:C) stimulation (Figure 3E).

### 3.3. Subcellular Localization of CiPrx3 Proteins

**Figure 4:**
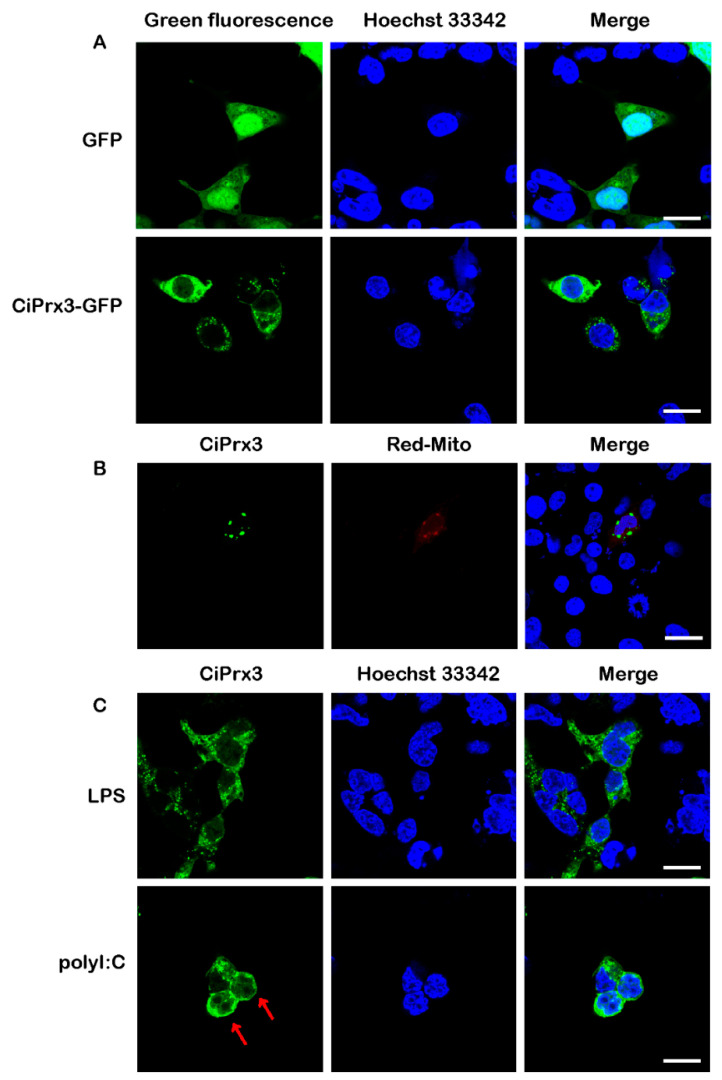
Subcellular localization of *CiPrx3* in GCO cells. (**A**) Subcellular localization patterns of *CiPrx3* protein in normal cells. (**B**) *CiPrx3*-EGFP colocalized with mitochondria. (**C**) Subcellular localization patterns of *CiPrx3* protein under PAMP stimulation. Red arrows indicate the nuclear translocation of the *CiPrx3* fusion protein induced by poly (I:C) stimulation. Scale bar = 20 μm.

In order to investigate the subcellular localization of the *CiPrx3* protein, GCO cells were transfected with pEGFP-*CiPrx3* and subjected to ﬂuorescence observation at 24 h post-transfection. pEGFP-N3 was transfected at the same time as a negative control. As shown in Figure 4A, green ﬂuorescence of the *CiPrx3*-EGFP fusion protein was found to be distributed only in the cytoplasm of transfected cells, while naked EGFP was distributed uniformly in whole cells. Moreover, pDsRed2-Mito, a mitochondria-specific marker vector, was co-transfected with pEGFP-*CiPrx3* to determine the precise localization of *CiPrx3*. Figure 4B shows that the green ﬂuorescence of *CiPrx3*-EGFP was colocalized with the red ﬂuorescence of mitochondria, suggesting *CiPrx3*-EGFP was localized in the mitochondria. In addition, the effects of PAMP stimulation on the subcellular localization of the *CiPrx3* protein were investigated. As shown in Figure 4C, subcellular localization patterns of *CiPrx3* protein were not changed after LPS stimulation, whereas *CiPrx3* protein seemed to concentrate in the nucleus after poly (I:C) stimulation, suggesting the nuclear translocation of *CiPrx3* proteins.

### 3.4. The Antioxidant Activity of CiPrx3

**Figure 5:**
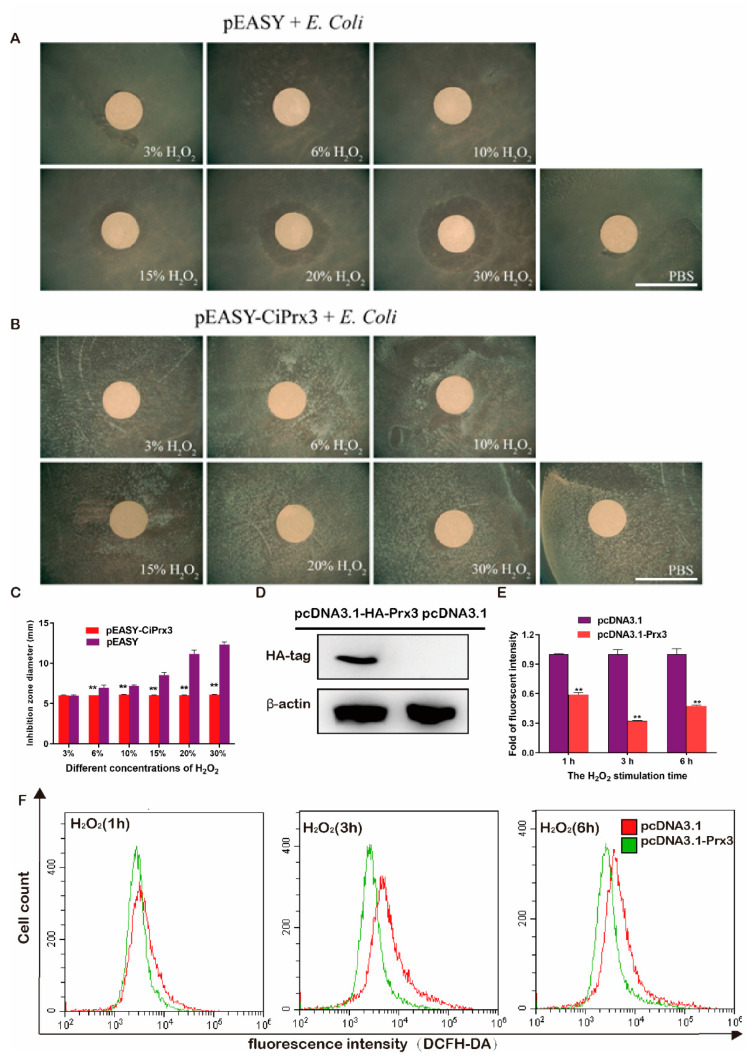
The antioxidant activity of *CiPrx3*. (**A**,**B**) *E. coli* BL21 strains were transformed with pEASY-Blunt or pEASY-*CiPrx3* and cultured in agarose plates treated with different concentrations of H_2_O_2_. Scale bar =1 cm. (**C**) Inhibition zone diameter of *E. coli* after H_2_O_2_ stimulation. (**D**) Confirmation of the overexpression of *CiPrx3* in GCO cells by Western blotting. GCO cells were transfected with pcDNA3.1-*CiPrx3* or pcDNA3.1 and harvested at 24 h for Western blotting by using an anti-HA antibody. (**E**) Calculation of relative fluorescence intensity of ROS in pcDNA3.1-*CiPrx3* or pcDNA3.1 transfected cells. (**F**) *CiPrx3* reduced the intracellular ROS caused by H_2_O_2_ stimulation. GCO cells were transfected with pcDNA3.1-*CiPrx3* or pcDNA3.1 and then treated with H_2_O_2_ (0.4 mM) and collected at different time points (1 h, 3 h, and 6 h) to detect the intracellular ROS by flow cytometry. Data were shown as the mean ± SD (*n* = 3). Asterisks represent significant differences (** = *p* ≤ 0.01).

*E. coli* BL21 strains were transformed with plasmids pEASY-*CiPrx3* or pEASY-Blunt, cultured in agarose plates, and then stimulated by different concentrations of H_2_O_2_ to test the antioxidant activity of *CiPrx3*. As shown in Figure 5, the growth of the pEASY-Blunt transformed strain was significantly inhibited when compared with the pEASY-*CiPrx3* transformed strain. The inhibition zone diameter of the pEASY-*CiPrx3*-transformed strain was significantly smaller than that of the control group under each concentration of H_2_O_2_ except for the 3% H_2_O_2_ (Figure 5A,B). To be specific, the diameter values of the pEASY-*CiPrx3* transformed strain were 1.00-, 0.87-, 0.85-, 0.71-, 0.54-, and 0.50-fold to that of control group under H_2_O_2_ stimulation at concentrations of 3%, 6%, 10%, 15%, 20%, and 30%, respectively (Figure 5C). Furthermore, GCO cells were transfected with pcDNA3.1-*CiPrx3* or pcDNA3.1 and then treated with H_2_O_2_ (0.4 mM) and collected at different time points (1 h, 3 h, 6 h) to detect the intracellular ROS by flow cytometry. The overexpression of *CiPrx3* in transfected cells was confirmed by Western blotting using an anti-HA antibody (Figure 5D). As shown in Figure 5E,F, the fluorescence intensity of ROS in pcDNA3.1-*CiPrx3* transfected cells was significantly lower than that of pcDNA3.1 transfected cells in all of the examined time points.

**Figure 6:**
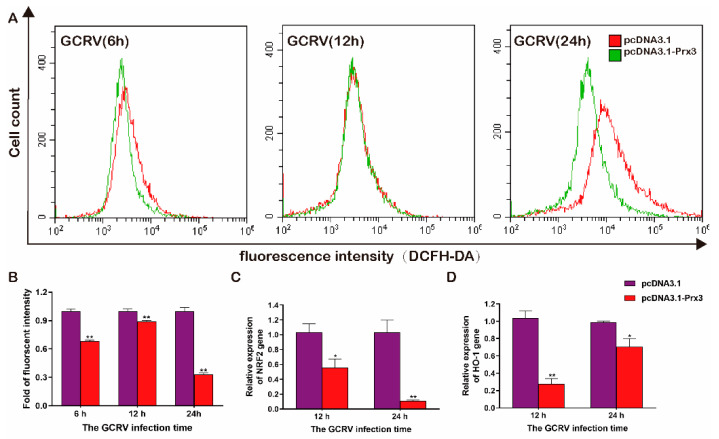
*CiPrx3* reduced the ROS caused by GCRV infection. (**A**) Flow cytometry assayed the intracellular ROS caused by GCRV infection. GCO cells were transfected with pcDNA3.1-*CiPrx3* or pcDNA3.1 and then infected with GCRV and collected at different time points (6 h, 12 h, and 24 h) to detect the intracellular ROS by flow cytometry. (**B**) The calculated relative fluorescence intensity of GCO cells. (**C**,**D**) The mRNA expression levels of nuclear factor erythroid 2-related factor 2 (NRF2) and heme oxygenase-1 (HO-1). Data were shown as the mean ± SD (*n* = 3). Asterisks represent significant differences (* = *p* ≤ 0.05, ** = *p* ≤ 0.01).

Moreover, it was proposed that virus infection could cause the accumulation of ROS in cells and thus induce cell damage and apoptosis [28]. Therefore, we investigated the antioxidant effect of *CiPrx3* during virus infection. GCO cells were transfected with pcDNA3.1-Prx3 or pcDNA3.1, then infected with GCRV. Cells were collected at different time points. Flow cytometry indicated that the intracellular ROS in pcDNA3.1-*CiPrx3* transfected cells were remarkably lower than those in the control group, especially in the 24 hpi (Figure 6A,B). Interestingly, we observed that the mRNA expression levels of two typical antioxidant genes, nuclear factor erythroid 2-related factor 2 (NRF2) and heme oxygenase-1 (HO-1), were decreased in the pcDNA3.1-Prx3 transfected cells when compared with the control cells (Figure 6C,D). Similar trends were also observed for other members of the Prxs family (Figure S3). Collectively, these results suggest the antioxidant activity of *CiPrx3*.

### 3.5. The Heavy Metal-Resistant and DNA Protection Ability of CiPrx3 

**Figure 7:**
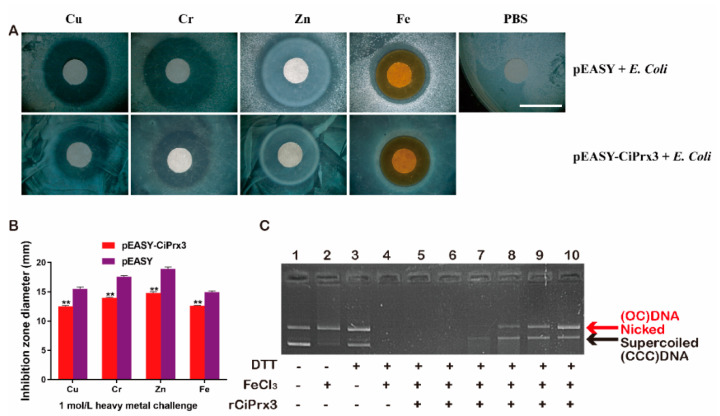
The heavy metal-resistant and DNA protection ability of *CiPrx3.* (**A**) The heavy metal-resistant ability of *CiPrx3*. *E. coli* BL21 strains were transformed with the pEASY-*CiPrx3* vector or pEASY-Blunt and then treated with different kinds of heavy metal ions. Scale bar = 1 cm. (**B**) Inhibition zone diameter of *E. coli* after heavy metal stimulation. Data were shown as the mean ± SD (*n* = 3). Asterisks represent significant differences (** = *p* ≤ 0.01). (**C**) Determination of the DNA protection ability of *CiPrx3* by MFO assay; 1: pcDNA3.1 without incubation; 2: pcDNA3.1 + FeCl_3_ (10 μM); 3: pcDNA3.1 + DTT (10 mM); 4: pcDNA3.1 + MFO mix (10 μM FeCl_3_ + 10 mM DTT); 5: pcDNA3.1 + MFO mix + 5 ng of r*CiPrx3*; 6: pcDNA3.1 + MFO mix + 50 ng of r*CiPrx3*; 7: pcDNA3.1 + MFO mix + 500 ng of r*CiPrx3*; 8: pcDNA3.1 + MFO mix + 1 μg of r*CiPrx3*; 9: pcDNA3.1 + MFO mix + 2 μg of r*CiPrx3*; 10: pcDNA3.1 + MFO mix + 4 μg of r*CiPrx3*. OC DNA: open circular plasmid DNA; CCC: covalently closed circular DNA.

The role of *CiPrx3* after exposure to different kinds of heavy metals was investigated. As shown in Figure 7A, the inhibition zone diameters of the pEASY-*CiPrx3*-transformed strains were markedly smaller than those of the control group under heavy metal ion Cu, Cr, Zn, and Fe treatment (Figure 7A). Specifically, the inhibition zone diameters of the pEASY-*CiPrx3*-transformed group were 0.81-fold, 0.80-fold, 0.78-fold, and 0.85-fold to those of the pEASY-Blunt-transformed group, respectively (Figure 7B). Collectively, these results indicate that *CiPrx3* enhanced host resistance to heavy metals. 

The recombinant *CiPrx3* protein was purified and confirmed by Western blotting (Figure S2). Then, an MFO assay was carried out in order to investigate whether the *CiPrx3* protein could prevent DNA damage caused by the ROS generated from an MFO system. As shown in Figure 7C, only FeCl_3_ could cause damage to the supercoiled DNA (Figure 7C, line 2), and the DNA damage was more serious when both DTT and FeCl_3_ existed (Figure 7C, line 4). Nevertheless, DNA damage was inhibited when the r*CiPrx3* protein existed, and the inhibitory effects were dose-dependent (Figure 7C, lanes 5–10), suggesting the DNA protection ability of *CiPrx3*.

### 3.6. The Anti-Apoptosis Ability of CiPrx3

**Figure 8:**
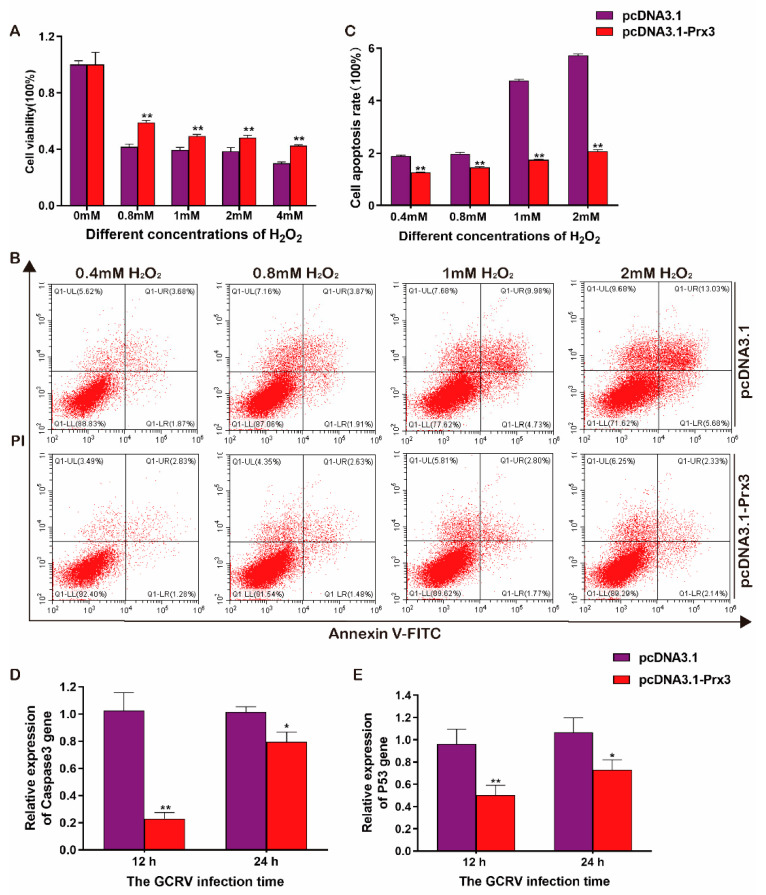
The anti-apoptosis ability of *CiPrx3*. (**A**) Cell viability of GCO cells after being treated with different concentrations of H_2_O_2_. (**B**) Flow cytometry assay of cell apoptosis of GCO cells after being treated with different concentrations of H_2_O_2_. GCO cells were transfected with pcDNA3.1-*CiPrx3* or pcDNA3.1 and stimulated by different concentrations of H_2_O_2_. Cells were collected to detect cell apoptosis by flow cytometry. (**C**) Calculated cell apoptosis rate of GCO cells after being treated with different concentrations of H_2_O_2_. (**D**,**E**) The relative mRNA expression level of Caspase 3 and p53. GCO cells were transfected with pcDNA3.1-*CiPrx3* or pcDNA3.1 and then infected with GCRV. Cells were harvested at different time points post-infection and the expression of apoptosis-related genes caspase 3 and p53 was detected by RT-qPCR. Data were shown as the mean ± SD (*n* = 3). Asterisks represent significant differences (* = *p* ≤ 0.05, ** = *p* ≤ 0.01).

In order to further investigate the function of *CiPrx3* during apoptosis, cells were transfected with pcDNA3.1-*CiPrx3* or pcDNA3.1 for overexpression and then treated with different concentrations of H_2_O_2_. The CCK-8 and flow cytometry assay indicated that the cell viability of the pcDNA3.1-*CiPrx3* transfected group was significantly higher than (Figure 8A), while the cell apoptosis rate was significantly lower than those of the pcDNA3.1 transfected group (Figure 8B,C). Moreover, the transfected cells were infected with GCRV and then harvested at 12 and 24 hpi in order to investigate expression patterns of apoptosis-related genes. Results revealed that both apoptosis-related genes, Caspase 3 and p53, showed significantly lower expression levels in pcDNA3.1-*CiPrx3* transfected cells when compared with the pcDNA3.1 transfected cells (Figure 8D,E). These results suggest the anti-apoptosis ability of *CiPrx3.*

### 3.7. CiPrx3 Inhibits GCRV Replication

**Figure 9:**
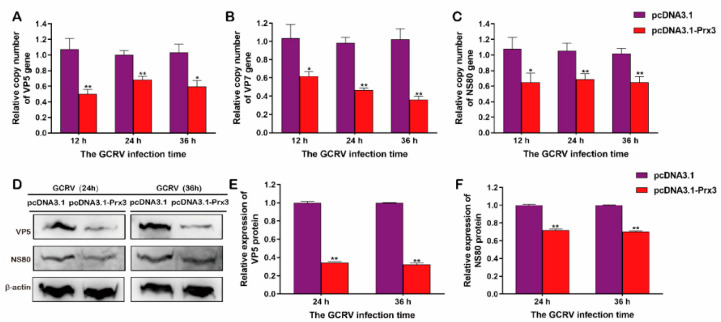
The anti-viral effect of *CiPrx3*. (**A**–**C**) Relative mRNA expression level of VP5 (**A**), VP7 (**B**), and NS80 (**C**). GCO cells were transfected with pcDNA3.1-*CiPrx3* or pcDNA3.1 and then infected with GCRV Cells were harvested at different time points post-infection for RT-qPCR analysis. (**D**) Western blotting analysis of the anti-viral effect of *CiPrx3*. GCO cells were transfected with pcDNA3.1-*CiPrx3* or pcDNA3.1 and then infected with GCRV. Cells were harvested at different time points post-infection for WB analysis. (**E**,**F**) Calculated protein expression levels of VP5 (E) and NS80 (**F**) at different time points post-infection. Data were shown as the mean ± SD (*n* = 3). Asterisks represent significant differences (* = *p* ≤ 0.05, ** = *p* ≤ 0.01).

In order to further investigate the role of *CiPrx3* during virus infection; GCO cells were transfected with pcDNA3.1-*CiPrx3* or pcDNA3.1 and then infected with GCRV, and cells were harvested at different time points. The mRNA and protein expression levels of viral non-structural protein NS80 and structural protein VP5 and VP7 in two groups were examined. RT-qPCR showed that mRNA expression levels of all three genes in pcDNA3.1-*CiPrx3* transfected cells were significantly lower than those in pcDNA3.1 transfected cells (Figure 9A–C). Moreover, WB analysis also revealed similar trends, in which protein expression levels of both NS80 and VP5 in pcDNA3.1-*CiPrx3* transfected cells were markedly lower than those of the controls (Figure 9D–F). These results indicated that *CiPrx3* inhibits GCRV replication.

### 3.8. CiPrx3 Promotes Autophagy to Inhibit GCRV Replication

**Figure 10:**
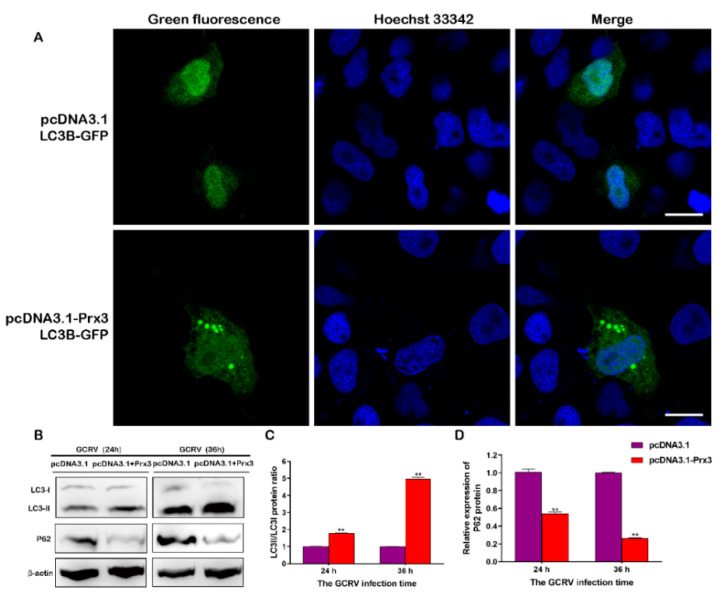
Overexpression of *CiPrx3* enhanced autophagy. (**A**) *CiPrx3* induced the accumulation of GFP-LC3B puncta in transfected cells. GCO cells were co-transfected with autophagy report plasmids GFP-LC3B and pcDNA3.1-*CiPrx3* or pcDNA3.1 for fluorescence observation. Scale bar = 20 μm. (**B**) *CiPrx3* enhanced autophagy after GCRV infection. GCO cells were transfected with pcDNA3.1 or pcDNA3.1-*CiPrx3* and then infected with GCRV; cells were harvested at different time points post-infection and protein expression levels of LC3-II/LC3-I and P62 were detected by Western blotting. (**C**,**D**) The calculated protein expression levels of LC3-II/LC3-I (**C**) and P62 (**D**) at different time points post GCRV infection. Data were shown as the mean ± SD (*n* = 3). Asterisks represent significant differences (** = *p* ≤ 0.01).

Some studies have reported that peroxiredoxins activate autophagy while it is important during host defense against pathogen infection [27,29,30]. Therefore, we investigate whether *CiPrx3* could induce autophagy and subsequently defend against GCRV infection. GCO cells were co-transfected with autophagy report plasmids GFP-LC3B and pcDNA3.1-*CiPrx3* or pcDNA3.1 for fluorescence observation. As shown in Figure 10A, the fluorescence of GFP-LC3 was distributed in the whole cells in pcDNA3.1 transfected cells, whereas pcDNA3.1-*CiPrx3* induced the accumulation of GFP-LC3 puncta in the cytoplasm, suggesting the induction of autophagy. Moreover, WB analysis showed that pcDNA3.1-*CiPrx3* significantly enhanced LC3-II expression and increased the ratio of LC3-II/LC3-I, but decreased the level of autophagy substrate P62 (Figure 10B–D). Altogether, these results indicated that *CiPrx3* promotes autophagy.

**Figure 11:**
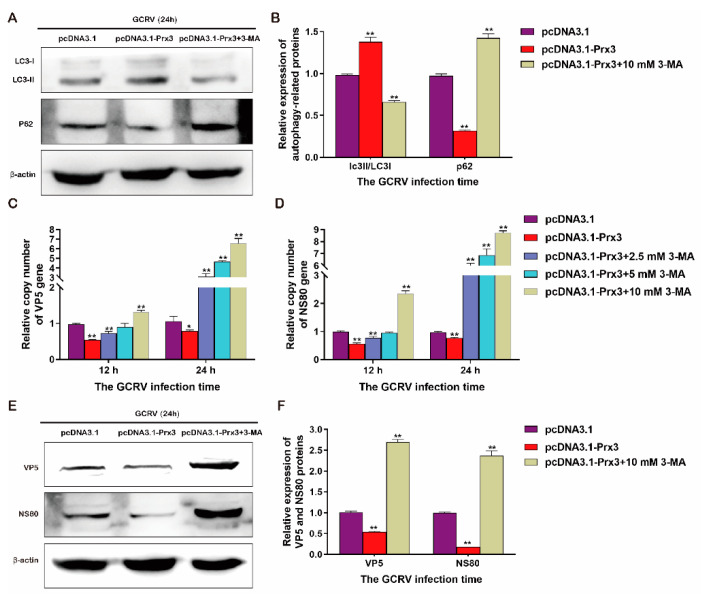
CiPrx3 enhanced autophagy to defend against GCRV. (**A**) The enhanced autophagy level induced by *CiPrx3* was blocked by 3-MA. Cells were transfected with pcDNA3.1 or pcDNA3.1-*CiPrx3* and treated with 3-MA, then were infected with GCRV and harvested at 24 h post-infection. Protein expression levels of LC3-II/LC3-I and P62 were detected by Western blotting. (**B**) The calculated protein expression levels of LC3-II/LC3-I and P62 at 24 h post-infection. (**C**,**D**) Relative mRNA expression of VP5 (**C**) and NS80 (**D**) in GCO cells. Cells were transfected with pcDNA3.1 or pcDNA3.1-*CiPrx3* and treated with different concentrations of 3-MA, then were infected with GCRV and harvested at different time points post-infection for RT-qPCR analysis. (**E**) The inhibition of GCRV replication by *CiPrx3* was blocked by 3-MA. Cells were transfected with pcDNA3.1 or pcDNA3.1-*CiPrx3* and treated with 3-MA, then were infected with GCRV and harvested at 24 h post-infection. Protein expression levels of VP5 and NS80 were detected by Western blotting. (**F**) The calculated protein expression levels of VP5 and NS80 at 24 h post-infection. Data were shown as the mean ± SD (*n* = 3). Asterisks represent significant differences (* = *p* ≤ 0.05, ** = *p* ≤ 0.01).

In order to confirm whether the inhibition of GCRV replication by *CiPrx3* is dependent on autophagy, cells were treated with 3-MA, a classic autophagy inhibitor, followed by plasmids transfection and GCRV infection. Cells were harvested at different time points after infection. As shown in Figure 11A,B, as expected, overexpression of *CiPrx3* promoted autophagy, whereas the enhanced autophagy level induced by *CiPrx3* was blocked by 3-MA, and appeared as a decreased LC3-II/LC3-I ratio and an increased level of P62 (Figure 11A,B). Moreover, after being treated with 3-MA, both the mRNA and protein expression levels of NS80 and VP5 were increased when compared with untreated cells (Figure 11C–F). Collectively, these results implied that *CiPrx3* promotes autophagy to inhibit GCRV replication.

## 4. Discussion

ROS, such as hydrogen peroxide (H_2_O_2_), hydroxyl radicals (HO^•^), and superoxidation (O_2_^•−^), are mainly produced by mitochondria as a result of an aerobic respiration process [31]. Normal cellular functions require low levels of ROS, which are involved in intracellular signal transduction [32], regulation of gene expression [33], host defense against pathogenic infections [34], and so on. Nevertheless, the mass accumulation of ROS might lead to nonspecific damage to proteins, lipids, and nucleic acids, and is associated with various diseases, such as cancers, cardiovascular diseases, and neurological diseases [35]. The balance between the production and elimination of ROS is therefore one of the most important topics in cell biology and physiology [36]. Peroxiredoxins have been identified in various organisms ranging from prokaryotes to eukaryotes, playing an important role in protecting organisms against oxidative stress [37]. In fish, reports on peroxiredoxins mainly focused on Prx1 and Prx2 [26,38], while information about Prx3 is limited. Herein, the Prx3 gene from grass carp was cloned, and its role in antioxidant activity and GCRV replication was analyzed.

Sequence analysis and multiple alignments revealed that Prx3 proteins were highly conserved in both fish and mammals, especially the two peroxiredoxin signature motifs (FYPLDFTFVCPTEI and GEVCFA) and the two highly conserved cysteines (Cys103 and Cys224), suggesting the important role of Prx3 during evolution. The two conserved Cys residues are required for their catalytic function, indicating that Prx3 could act as a reductant to reduce ROS production during cell metabolism and oxidative stress [7]. *CiPrx3* showed the most similar identity to Prx3 from *M. piceus*, *C. carpio*, and *D. rerio*. Moreover, the phylogenetic tree implied that *CiPrx3* was clustered into the same clade as the Prx3 class in teleost fish. All of the results suggest that *CiPrx3* belongs to the typical 2-Cys Prx subfamily.

In all examined tissues, *CiPrx3* was constitutively expressed with different expression levels. Relatively higher expression levels were found in the liver and muscle, which are important organs for metabolic processes and energy production [39,40]. Mitochondria are considered energy factories of cells and should therefore be abundant in the liver and muscle for energy production [41]. A large number of mitochondria in the liver and muscle may cause the accumulation of ROS [42,43,44]. Therefore, the higher expression level of *CiPrx3* in the liver and muscle may be beneficial for reducing the accumulation of ROS. Highly expressed levels of *Prx3* in liver or muscle tissues were also observed in other fish, such as medaka [15], gilthead sea bream [45], rock bream [16], and large-belly seahorses [5], implying the conserved role of Prx3. 

The antioxidant activity of *CiPrx3* was determined by transforming the prokaryotic vector pEASY-*CiPrx3* into *E. coli* BL21 strains or transfecting the eukaryotic vector pcDNA3.1-CiPrx3 into GCO cells. Both were then treated with H_2_O_2_. The stains on cells with pcDNA3.1-*CiPrx3* presented significant growth activity, cell viability, and antiapoptotic activity compared with controls, suggesting the antioxidant activity of *CiPrx3* [14,46,47]. Moreover, MFO assays showed that the r*CiPrx3* protein could inhibit DNA damage in a dose-dependent manner, further indicating the antioxidant activity of *CiPrx3*. Similar results of Prxs-mediated inhibition of DNA damage caused by the MFO system were also reported in other fish species [5,48]. Interestingly, we also found that the *E. coli* BL21 strains with *CiPrx3* also showed resistance to heavy metal toxicity, which illustrated the important role of *CiPrx3* not only in the host response to oxidative stress, but also in the response to heavy metal toxicity.

It was reported that GCRV infection could cause the accumulation of ROS in cells [49]. Overexpression of *CiPrx3* in fish cells remarkably reduced intracellular ROS caused by GCRV infection, further suggesting the antioxidant activity of *CiPrx3*. Surprisingly, after overexpression of *CiPrx3* in GCO cells, we observed reduced mRNA expression levels of other antioxidant genes, such as NRF2, HO-1, and other members of Prxs family. The clarity of abundant ROS in organisms requires the cooperation of many enzymes, such as superoxide dismutase (SOD), catalase (CAT), glutathione peroxidase (GSH-Px), and peroxiredoxins (Prxs) [35]. NRF2 is a transcription factor that activates the oxidative stress defense system by inducing antioxidant and detoxifying enzymes to protect cells from oxidative damage [50]. HO-1 and peroxiredoxins are the target genes that are regulated by NRF2 [51,52]. Thus, it could be speculated that overexpression of *CiPrx3* reduced intracellular ROS and resulted in decreased levels of NRF2, as well as its target genes, such as HO-1 and peroxiredoxins.

Regarding the peroxiredoxin family, Prx1 and Prx2 were reported to be involved in the fish immune response [1,53], while it is still unclear whether Prx3 participates in the immune response. The expression of *CiPrx3* was significantly altered after exposure to GCRV and PAMPs, indicating that *CiPrx3* may be involved in the immune response. In order to reveal the specific role of *CiPrx3* during virus replication, GCO cells were transfected with pcDNA3.1 and pcDNA3.1-*CiPrx3* for overexpression, and then infected with GCRV. Both RT-qPCR and Western blotting results indicated that *CiPrx3* inhibits GCRV replication. Interestingly, we also observed the induction of autophagy by *CiPrx3* along with the inhibition of GCRV replication, prompting us to speculate whether the inhibition dependent on autophagy. Therefore, cells were treated with autophagy inhibitor 3-MA, followed by transfection and GCRV infection. As expected, the enhanced autophagy level induced by *CiPrx3* was blocked by 3-MA. Meanwhile, we also observed decreased mRNA and protein expression levels of viral genes after being treated with 3-MA. Therefore, these results indicated that the imbibition of GCRV replication by *CiPrx3* is dependent on autophagy.

## 5. Conclusions

In conclusion, peroxiredoxin 3, a member of the typical 2-Cys Prxs subfamily, was cloned from grass carp. *CiPrx3* was localized in the mitochondria of transfected cells and concentrated in the nucleus after poly (I:C) treatment. *CiPrx3* enhanced the resistance of *Escherichia coli* to H_2_O_2_ and heavy metals, and reduced intracellular ROS in fish cells after H_2_O_2_ treatment and GCRV infection. Purified recombinant *CiPrx3* protein could protect DNA against oxidative damage. Furthermore, *CiPrx3* could induce autophagy and subsequently inhibit GCRV replication in fish cells. Our study provides more information for gaining further understanding of the immune function of Prx3 in teleost fish.

## Data Availability

The data presented in this study are available in the article.

## Supplementary Materials

The following supporting information can be downloaded at: https://www.mdpi.com/article/10.3390/antiox11101952/s1, Figure S1: Sequence analysis of CiPrx3; Figure S2: Prokaryotic expression and Western blotting analysis of *CiPrx3*; Figure S3: The effect of *CiPrx3* overexpression on mRNA expression of other members from Prxs family; Table S1: Primer sequences used in the study.
