# Supplementary material for: Grass Carp Prx 3 Elevates Host Antioxidant Activity and Induces Autophagy to Inhibit Grass Carp Reovirus (GCRV) Replication
Source: Antioxidants (Basel). 2022 Sep 29;11(10):1952. doi: 10.3390/antiox11101952 (PMC9598204; doi:10.3390/antiox11101952)
Supplement: Supplementary file 1 [file antioxidants-11-01952-s001.zip › Supplemental Figure S1.pdf]

A

```
1                 M  A  A  T  I  G  R  L  L  G  T  S  A  R  R  A  A  V  N  G  L
1    gcacattggcaagATGGCAGCCACCATCGGGAGACTTCTCGGGACTTCTGCAAGAAGAGCTGCAGTTAATGGACT
22    K  V  L  V  P  H  N  G  P  S  V  I  R  A  P  Q  S  L  A  C  I  A  A  Q  K
76   GAAGGTCTTGGTCCCTCACAATGGACCATCTGTGATCAGGGCCCCACAATCCCTTGCCTGCATTGCTGCACAAAA
47    A  C  F  S  V  S  T  A  R  W  A  A  A  V  T  Q  Q  A  P  H  F  K  G  T  A
151  AGCTTGCTTCTCAGTCAGCACTGCTAGATGGGCTGCAGCAGTCACTCAACAGGCCCCACATTTCAAAGGCACTGC
72    V  L  N  G  E  F  K  E  I  S  L  D  D  F  K  G  K  Y  L  V  L  F  F  Y  P
226  TGTTCTCAATGGAGAGTTCAAGGAGATCAGCCTAGATGATTTCAAGGGCAAATACCTGGTCCTTTTCTTTTACCC
97    L  D  F  T  F  V  C  P  T  E  I  I  A  F  S  D  K  A  N  E  F  R  D  V  N
301  ACTAGATTTCACTTTTGTTTGCCCCACAGAGATCATCGCCTTCAGTGACAAAGCCAATGAGTTTCGTGATGTAAA
122   C  Q  V  V  G  V  S  V  D  S  H  F  T  H  L  A  W  T  N  T  P  R  K  S  G
376  CTGTCAAGTAGTTGGTGTGTCTGTGGACTCTCACTTTACCCACCTGGCCTGGACCAACACCCCAAGAAAGAGTGG
147   G  L  G  K  I  H  I  P  L  L  A  D  L  T  K  Q  V  S  R  D  Y  G  V  L  L
451  AGGATTAGGGAAAATCCACATTCCCCTGTTGGCTGATCTCACAAAGCAAGTGTCCAGAGACTATGGGGTCCTGCT
172   E  G  P  G  I  A  L  R  G  L  F  I  I  D  P  N  G  V  I  R  H  M  S  V  N
526  GGAGGGTCCTGGAATTGCTCTAAGGGGTCTTTTCATTATTGATCCTAATGGAGTGATCCGACACATGAGTGTAAA
197   D  L  P  V  G  R  S  V  E  E  T  L  R  L  V  K  A  F  Q  F  V  E  T  H  G
601  TGACCTGCCGGTTGGACGCTCTGTTGAGGAAACCCTCCGTCTGGTCAAGGCCTTCCAGTTTGTGGAAACCCATGG
222   E  V  C  P  A  S  W  T  P  K  S  P  T  I  K  P  T  P  D  G  S  K  E  Y  F
676  TGAAGTCTGTCCTGCCAGCTGGACCCCAAAATCACCCACGATTAAGCCGACTCCAGATGGCTCAAAGGAATACTT
247   E  K  V  N  *
751  TGAGAAGGTCAACTGAaaaccctaaaactattggtactgacagatcctgcagatcttggcatttaactgaaacaa
826  gtccataatccatatccaaaatccaccatttatagacttaatgtatcagtatgttagcagttaatagggagtgga
901  atctgttttgatttatgtatttaagctgttcaaataaaaaccgcactgttaaatttgtctctttgccatagtaca
976  tgctttctatccgaagtgcttcactctctgacataagcaaataatttgtgactctttgaataaatattgtgtaat
1051 tgtataaaaatatatgca
```

B

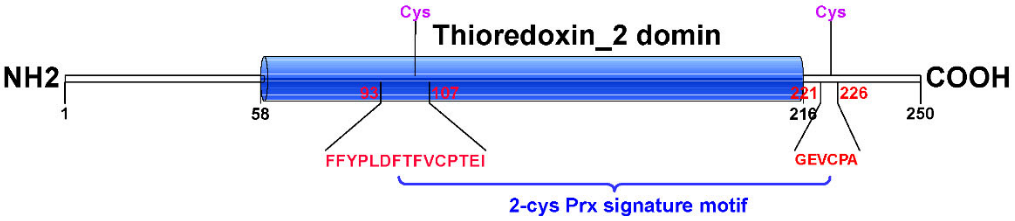

Supplemental Figure S1. Sequence analysis of CiPrx3. (**A**) Nucleotide and deduced amino acid sequences of *CiPrx3*. The nucleotide sequence (lower) and the deduced amino acid sequence (upper) were numbered. Start codon (ATG) and the stop codon (TAA) were highlighted by yellow color. The thioredoxin domain was colored in dark green shaded and Prx signature motifs (FFYPLDFTFVCPTEI and GEVCPA) were in red. The conserved active cysteines were boxed. (**B**) The functional domain architecture of CiPrx3 protein.
