# Supplementary material for: Grass Carp Prx 3 Elevates Host Antioxidant Activity and Induces Autophagy to Inhibit Grass Carp Reovirus (GCRV) Replication
Source: Antioxidants (Basel). 2022 Sep 29;11(10):1952. doi: 10.3390/antiox11101952 (PMC9598204; doi:10.3390/antiox11101952)
Supplement: Supplementary file 1 [file antioxidants-11-01952-s001.zip › Supplemental Figure S2.pdf]

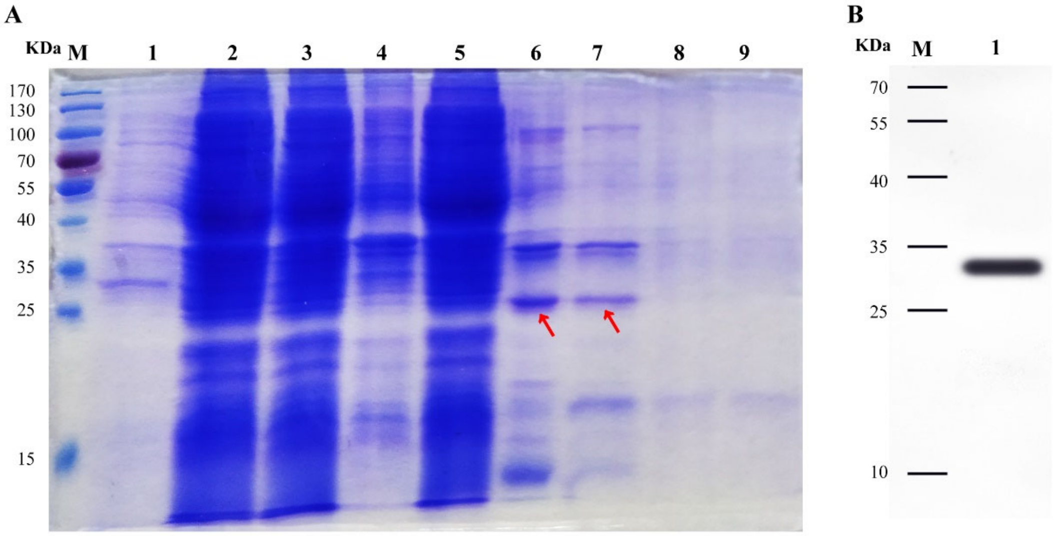

Supplemental Figure S2. Prokaryotic expression and Western blotting analysis of CiPrx3. (**A**) Prokaryotic expression of CiPrx3 recombinant protein. Lane M: Protein molecular weight marker; lane 2: whole bacterial lysate after 6 h induction with 1.0 mM IPTG; lane 3: supernatant of lysate; lane 4, precipitation of lysate; lane 5, the passed lysate through Ni-NTA resin; lane 5-9, recombinant CiPrx3 protein eluted from Ni-NTA resin with different concentrations (80 mM, 120 mM, 200 mM and 500 mM) of imidazole. (**B**) Western blot analysis of recombinant CiPrx3 proteins with anti-His-tag antibody. Lane M: protein molecular weight marker; lane 1: purified recombinant CiPrx3 protein.
