# Supplementary material for: Grass Carp Prx 3 Elevates Host Antioxidant Activity and Induces Autophagy to Inhibit Grass Carp Reovirus (GCRV) Replication
Source: Antioxidants (Basel). 2022 Sep 29;11(10):1952. doi: 10.3390/antiox11101952 (PMC9598204; doi:10.3390/antiox11101952)
Supplement: Supplementary file 1 [file antioxidants-11-01952-s001.zip › Supplemental Figure S3.pdf]

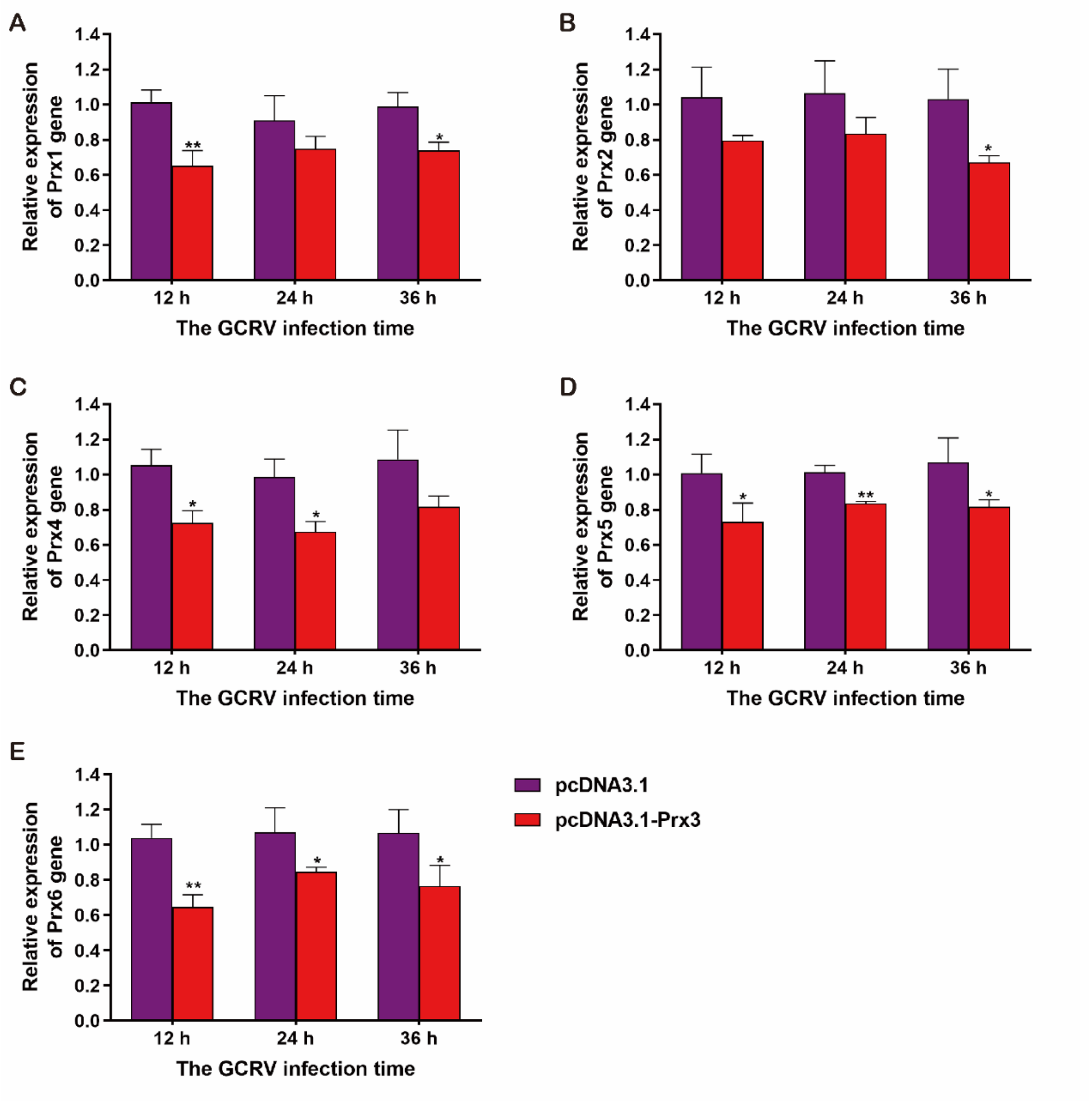

Supplemental Figure S3. the antioxidant effect of CiPrx3 on prx3 subfamily genes during virus infection. (**A-E**) Relative mRNA expression level of Prxs subfamily genes. GCO cells were transfected with pcDNA3.1-Prx3 or pcDNA3.1 and then infected with GCRV and cells were collected at different time points. Data were shown as the mean ± SD (n = 3). Asterisks (*) represent significant differences (* = $p \leq 0.05$, ** = $p \leq 0.01$).
