# Supplementary material for: Grass Carp Prx 3 Elevates Host Antioxidant Activity and Induces Autophagy to Inhibit Grass Carp Reovirus (GCRV) Replication
Source: Antioxidants (Basel). 2022 Sep 29;11(10):1952. doi: 10.3390/antiox11101952 (PMC9598204; doi:10.3390/antiox11101952)
Supplement: Supplementary file 1 [file antioxidants-11-01952-s001.zip › Table S1.pdf]

Primers	Sequences (5'—3')	Purpose
Prx3-F	ATGGCAGCCACCATCGG	ORF cloning
Prx3-R	TCAGTTGACCTTCTCAAAGTATTCCTT	
Lc-3B-F	ATGCCTTCGGAAAAGACA	
Lc-3B-R	TTACTGAGGACACGCAGTTCC	
Prx3-5' out	TGATCTCCTTGAACTCTCCATTGAG	5' RACE of Prx3
Prx3-5' in	GTGCAGCAATGCAGGCAAGG	
Prx3-3' out	CTGCCGGTTGGACGCTCTG	3' RACE of Prx3
Prx3-3' in	TGGTGAAGTCTGTCCTGCCAG	
qPrx3-F	TAGTTGGTGTGTCTGTGGACTCTCA	RT-qPCR
qPrx3-R	TCTCTGGACACTTGCTTTGTGAGAT	
qβ-actin-F	TCGGTATGGGACAGAAGGAC	
qβ-actin-R	GACCAGAGGCATACAGGGAC	
qHO-1-F	CAGCATCCCAAAGTCAATCAAG	
qHO-1-R	GCATAAACTCCCATTCCAACAG	
qNrf2-F	GTGAATGAGGAGGAGGTGAAAG	
qNrf2-R	GGGTACTACTTCCCAGCAAATC	
qCaspase-3-F	TGACCAGGGTCAACCATAAAG	
qCaspase-3-R	TGGTGAGCATCGAGACAATG	
qp53-F	CCCATCCTCACAATCATCACTC	
qp53-R	TCTTGCTTGGGGTTTTGGTCTC	
qprx1-F	GCAAACCTGCTCCAGACTTCAC	
qprx1-R	CAAACAAAGGTGAAATCCAATGG	
qprx2-F	TCTACCCGCTTGATTTCACCTTC	
qprx2-R	AGCGATGACCTCACAGCCG	
qprx4-F	GAGTTCCAGGACATCAACGCAG	
qprx4-R	TGGTGAGTGAGGTCGGAAAGG	
qprx5-F	GACTCATCTCCCAGGGTTCGT	
qprx5-R	GCCATCTGCTCCGTTCTGTTTT	
qprx6-F	GCATCTTGTTCTCGCACCCAC	
qprx6-R	CATCTTCACGTCCCGTTTCTT	
pEGFP-LC3B-F	CGCTCGAGGTATGCCTTCGGAAAAGACA	Subcellular localization
pEGFP-LC3B-R	CGGGATCCCTGAGGACACGCAGTTCC	
pEGFP-Prx3-F	CGCTCGAGGTATGGCAGCCACCATCGG	
pEGFP-Prx3-R	CGGGATCCGTTGACCTTCTCAAAGTATTCCTT	
pEASY-Prx3-F	ATGTCTGCTGGAAACGCTAAGAT	Recombinant expression
pEASY-Prx3-R	TTACTGCTTGGAGAAGAACTCCTT	
